# Region-specific hamstring activation and its dissociation from subjective contraction sensation during leg curl exercise at different hip flexion angles

**DOI:** 10.1186/s13102-026-01910-7

**Published:** 2026-07-31

**Authors:** Hiroku Mitsuya, Koichi Nakazato, Takashi Okada

**Affiliations:** 1 Research Institute for Sport Science, Nippon Sport Science University, 7‑1‑1 Fukasawa, Setagaya‑ku, Tokyo, Japan; 2 Faculty of Medical Science, Nippon Sport Science University, 1221‑1 Kamoshida‑cho, Aoba‑ku, Yokohama‑shi, Kanagawa Japan; 3 Faculty of Sport Science, Nippon Sport Science University, Fukasawa, Setagaya‑ku, Tokyo, Japan

**Keywords:** Resistance training, Bodybuilding, Hamstring muscle, T2 activation rate

## Abstract

**Background:**

Hamstring activation during resistance training may vary according to muscle region and joint position. However, the relationship between region-specific hamstring activation and subjective contraction sensation during leg curl exercise remains unclear. The purpose of this study was to investigate region-specific and longitudinally compartmentalized muscle dynamics of the hamstring during leg curl (LC) exercise performed at different hip flexion angles (HFA), and to examine the relationship between subjective contraction sensation and objective muscle activity.

**Methods:**

Nine male bodybuilders performed unilateral LC at three hip flexion angles (0°, 40°, and 80°), with knee flexion ranging from 0° to 130°. Pre- and post-exercise transverse relaxation times (T2) were measured using magnetic resonance imaging. Each LC session consisted of 5 sets of 10 repetitions at 70% of one-repetition maximum, with 1-minute rest intervals. The hamstring was divided into proximal, middle, and distal regions for the biceps femoris long (BF-long) and short heads, semitendinosus (ST), and semimembranosus. T2 activation rates were calculated for each region. The subjective sensation of hamstring contraction during LC exercise was evaluated using a numerical rating scale (NRS) and compared with the T2 value.

**Results:**

Across all conditions, the ST exhibited greater activation than the other hamstring muscles (*p* < 0.01, *p* < 0.05). In particular, the distal region showed higher activation than the proximal region at HFA 0° (*p* < 0.05). For the BF-long, greater activation was observed in the proximal region compared with the distal region at HFA 40° (*p *< 0.01). The NRS scores were inconsistent with the objective index.

**Conclusion:**

The ST is preferentially activated during LC exercise. Hamstring activation is both region-specific and longitudinally compartmentalized, depending on muscle length as influenced by HFA. Because NRS was dissociated from T2 values, hamstring resistance training should not rely solely on perceived muscle contraction, but should instead consider objective muscle activation characteristics. However, since NRS and T2 values were not directly compared in the present study, further advanced research addressing these limitations is warranted in the future. Optimal hip positioning should therefore be considered when designing resistance training programs to effectively target specific hamstring regions.

**Graphical Abstract:**

**Figure:**
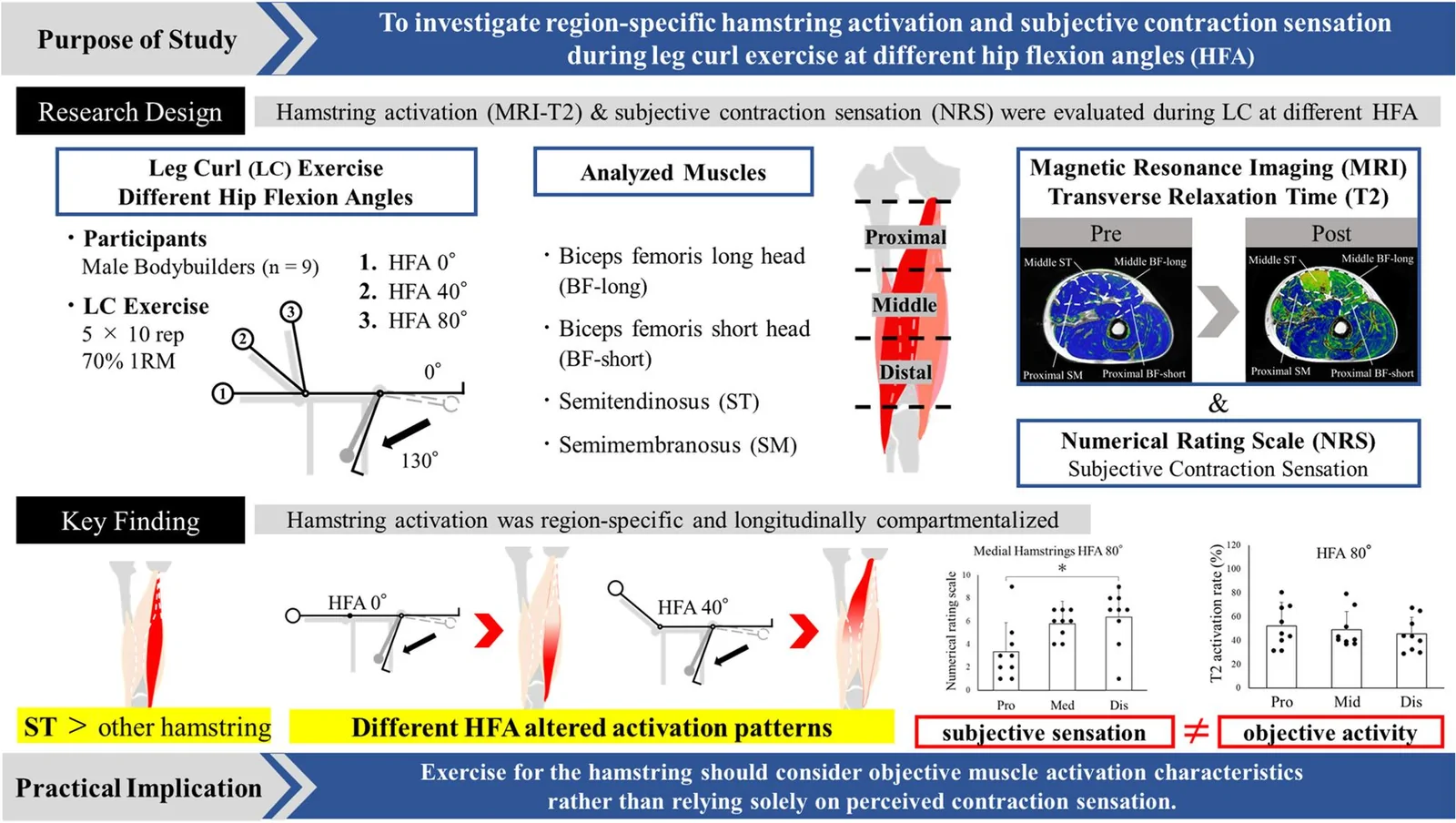

**Supplementary Information:**

The online version contains supplementary material available at https://doi.org/10.1186/s13102-026-01910-7.

## Highlights

Hip flexion angle modulates region-specific hamstring activation during leg curl exercise.

Semitendinosus exhibits consistently greater activation than other hamstring muscles across conditions.

Hip positioning enables selective targeting of distal semitendinosus and proximal biceps femoris long head during resistance training.

Subjective contraction sensation did not correspond with objective hamstring muscle activity under any leg curl condition.

**Supplementary Information:**

The online version contains supplementary material available at https://doi.org/10.1186/s13102-026-01910-7.

## Introduction

Skeletal muscles demonstrate region-specific activation patterns across different muscles within the same group, as well as longitudinally compartmentalized activation within a single muscle [1, 2]. Reports using magnetic resonance imaging (MRI) and electromyography have demonstrated significant regional differences in both muscle hypertrophy and muscle activation between muscles and within individual muscles [3–11]. In our previous research, we focused on the quadriceps femoris and investigated both region-specific and longitudinally compartmentalized muscle dynamics [5, 6]. During leg extension exercises performed at varying hip flexion angles (HFA), changes in HFA were found to alter muscle length [12], which in turn influenced both region-specific and longitudinal muscle activation patterns [5, 6]. One possible explanation for this phenomenon is the anatomical feature of the rectus femoris, which receives motor innervation from separate nerve branches in its proximal and distal regions [13]. One possible explanation for this phenomenon is that the proximal and distal regions of the rectus femoris are innervated by separate motor nerve branches, which may contribute to their region-specific activation patterns. This pattern of motor innervation is thought to contribute to both region-specific activation and longitudinally compartmentalized muscle dynamics.

The hamstring muscles comprise the biarticular biceps femoris long head (BF-long), semitendinosus (ST), semimembranosus (SM), and the monoarticular biceps femoris short head (BF-short). Similarly, the hamstring share anatomical features with the quadriceps, as the motor nerve branches of the BF-long are divided between the proximal and distal region [14]. Additionally, a tendinous inscription located near the midpoint of the ST separates the muscle belly into proximal and distal compartments [14]. In addition to the clear anatomical partitioning of the ST by its tendinous inscription, the motor nerve branches of the ST are also divided between the proximal and distal regions [14]. Consistent with this anatomical arrangement, previous studies have reported that electromyographic activity differs between the proximal and distal portions of the ST depending on joint angle [15, 16]. Furthermore, skeletal muscles have an optimal length for force production. Therefore, changes in joint angle alter muscle length, and the resulting differences in muscle length within a muscle may lead to region-specific variations in force-generating capacity [17]. These anatomical characteristics, as well as the pattern of motor innervation, may underlie distinct region-specific activation in each muscle head and contribute to longitudinally compartmentalized muscle dynamics within individual hamstring muscles.

Indeed, previous studies have shown that leg curl (LC) exercises at different HFAs elicit distinct hypertrophic responses in individual hamstring muscles. Prone LC performed in a lying position with an HFA of approximately 0°, primarily induces hypertrophy in the BF-long and ST [18], whereas seated LC at an HFA of approximately 80° leads to hypertrophy not only in the BF-long and ST but also in the SM [18]. Although numerous studies have investigated region-specific characteristics within the hamstring [19–22], the findings remain inconsistent, and many aspects are still unclear. In particular, evidence regarding longitudinally compartmentalized activation within individual hamstring remains lacking. Although this finding was not reported in the hamstring, previous studies have demonstrated that region-specific changes in MRI transverse relaxation time (T2) values following acute exercise correspond with region-specific hypertrophy observed after chronic training [8, 9]. Therefore, it cannot be ruled out that a similar relationship may also exist in the hamstring. A previous study by Watanabe et al. reported no evidence of longitudinally compartmentalized muscle activation in the BF-long [23]. However, as their findings were based on limited assessments using electromyography, our study extended this line of inquiry by employing MRI, thereby establishing a novel methodology and uncovering new insights [5].

The primary aim of this study was to investigate how changes in HFA influence region-specific and longitudinally compartmentalized muscle dynamics during LC. The T2 value obtained from MRI, which enables detailed analysis, is considered optimal for observing region-specific and longitudinally compartmentalized muscle dynamics [5, 7–9]. We hypothesized that modulation of HFA would induce changes in muscle length, leading to previously unconfirmed region-specific and longitudinally compartmentalized responses within the hamstring components. To ensure accurate and consistent execution of the training movements, the study recruited experienced male bodybuilders who were highly skilled in resistance training [5, 6]. Additionally, to obtain practical insights applicable to resistance training settings where objective assessment of muscle activity is difficult, the relationship between objective muscle activity and subjective sensations of muscle contraction was also investigated. By investigating the relationship between hamstring activation and subjective sensation, a more comprehensive understanding of hamstring function may be achieved. The findings of the present study may provide a basis for the development of novel training programs aimed at region-specific strengthening of the hamstring. Given that hamstring strain injuries are most frequently reported in the proximal region, particularly during high-speed running [24, 25], such approaches may ultimately contribute to a better understanding of the mechanisms underlying hamstring injuries [26–31], the establishment of injury prevention strategies, and the design of training programs to enhance sprint performance [32].

## Methods

### Participants

This study was approved by the Ethics Committee of Nippon Sport Science University (Approval No. 021-H211, January 31, 2022) and conducted in accordance with the Declaration of Helsinki [33]. All participants provided written informed consent before participation, and the purpose and potential risks of the study were fully explained to each subject. Nine male bodybuilders (age: 20.4 ± 1.2 years; height: 172.0 ± 3.9 cm; body weight: 73.1 ± 3.6 kg; bodybuilding training experience: 4.6 ± 1.2 years) participated. Participants with no history of thigh or knee injuries were included. All participants engaged in hypertrophy-oriented resistance training and were currently performing high-intensity split-body routines lasting approximately 1–3 h per session, at least four times per week.

### Measurement protocol

**Fig. 1:**
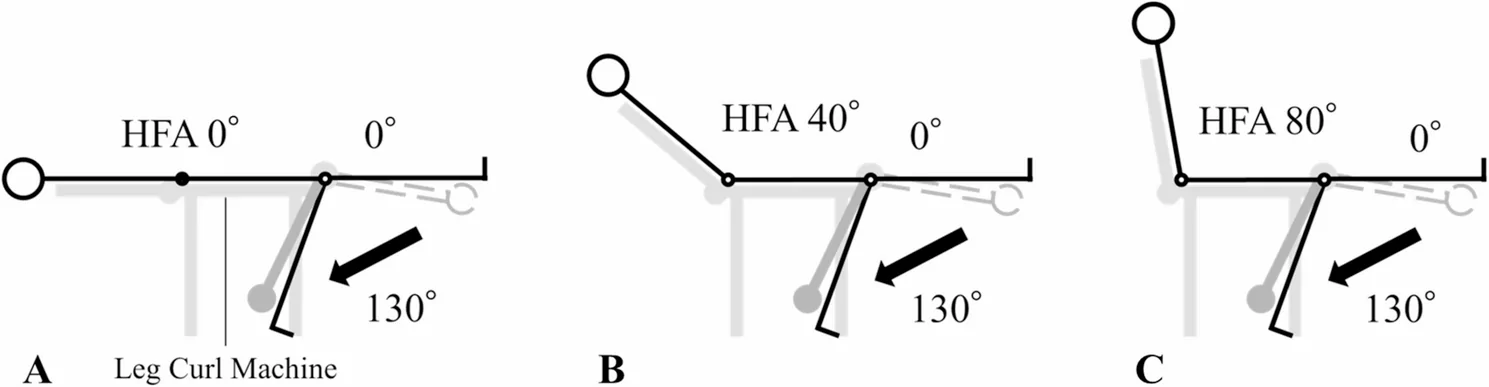
LC exercise under three HFA conditions using an LC machine. **A** HFA 0°: the maximum hip extension achievable with the machine settings. **B** HFA 40°: the intermediate angle between the three conditions. **C** HFA 80°: the conventional seated LC position. In all conditions, the knee joint was flexed from 0° (full extension) to 130° (maximum flexion). HFA, hip flexion angle

Unilateral LC exercises were performed using a leg extension and curl bench (IROTEC, Osaka, Japan), with knee flexion ranging from 0° (full extension) to 130°. The test leg was randomly selected for each participant. Because limb dominance was not a variable of interest in the present study, the tested leg was randomly assigned to avoid systematic bias related to dominant or non-dominant limb characteristics. Three HFAs were set: 0°, 40°, and 80°. An HFA of 80° represented the conventional seated LC position, 0° corresponded to the maximum hip extension achievable with the machine settings, and 40° served as an intermediate angle (Fig. 1). To ensure accuracy and consistency in the range of motion, a rubber band was positioned so that the frame of the machine contacted it when the knee reached 130° of flexion during each repetition. The pelvis was additionally secured to the machine using a non-elastic strap to prevent postural deviations during the trials.

**Fig. 2:**
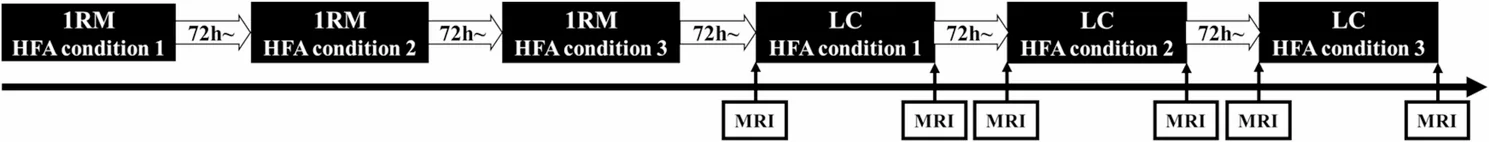
Experimental schedule for LC measurement. The 1RM for each of the three HFA conditions was measured at intervals of at least 72 h. Based on these 1RM values, LC sessions were performed with a minimum interval of 72 h between sessions. MRI scans were conducted both before and after each LC session. The order of 1RM testing and LC execution across the HFAs was randomized. 1RM, one-repetition maximum; LC, leg curl; MRI, magnetic resonance imaging

To determine the appropriate training load, the one-repetition maximum (1RM) for each of the three HFA conditions was assessed at least 72 h apart during the week preceding the MRI measurements. During the 1RM assessment, a trial was deemed unsuccessful if the participant could not maintain the prescribed movement tempo or could not achieve the predetermined range of motion. Based on the measured 1RM values, the LC exercise was performed at 70% of 1RM for 5 sets of 10 repetitions, with 1-minute rest intervals between sets. Although a 1-minute rest interval between sets may be relatively short, it was adopted in the present study to avoid recovery of blood flow dynamics and a consequent reduction in T2 values that may occur with longer rest periods. The HFA condition used for the measurement was randomly selected from the three. To minimize fatigue-related effects, at least 72 h were provided between each MRI measurement session. Care was taken to ensure that none of the measurement days overlapped with the participants’ habitual lower-limb resistance training sessions. The experimental schedule is illustrated in Fig. 2. All measurements were conducted by an investigator certified as a strength and conditioning specialist certified by the national strength and conditioning association.

### MRI measurement

Muscle activity was assessed by calculating T2 relaxation times using an MRI system (ECHELON OVAL; 1.5T, Hitachi Medical Systems, Tokyo, Japan). MRI scanning was performed using a protocol capable of capturing the entire thigh in a single acquisition under resting supine conditions. The imaging parameters were as follows: echo times = 25, 50, 75, and 100 ms; repetition time = 5000 ms; matrix = 512 × 320; field of view = 40 cm; number of slices = 25; slice thickness = 1 cm; gap = 1 cm. The entire scan was completed within 15 min. To ensure consistent positioning, a mark was placed on the skin at the midpoint of a line connecting the greater trochanter and lateral epicondyle of the femur using a permanent marker. This point was aligned with the laser crosshair from the MRI, which served as the central reference point of the scanned image. This alignment ensured that the 13th slice (the center of 25 slices) corresponded with the anatomical midpoint of the femur. To avoid fluctuations caused by preceding movement, the pre-LC (resting) scans were conducted after participants had rested in a supine position for at least 20 min upon arrival at the MRI facility. All measurements were conducted around noon, and no other physical activity was permitted on the day of assessment. The LC protocol (10 repetitions × 5 sets) was performed in the anteroom adjacent to the MRI scanner, and post-exercise imaging was initiated within one minute after completion of the LC session.

### MRI calculation

**Fig. 3:**
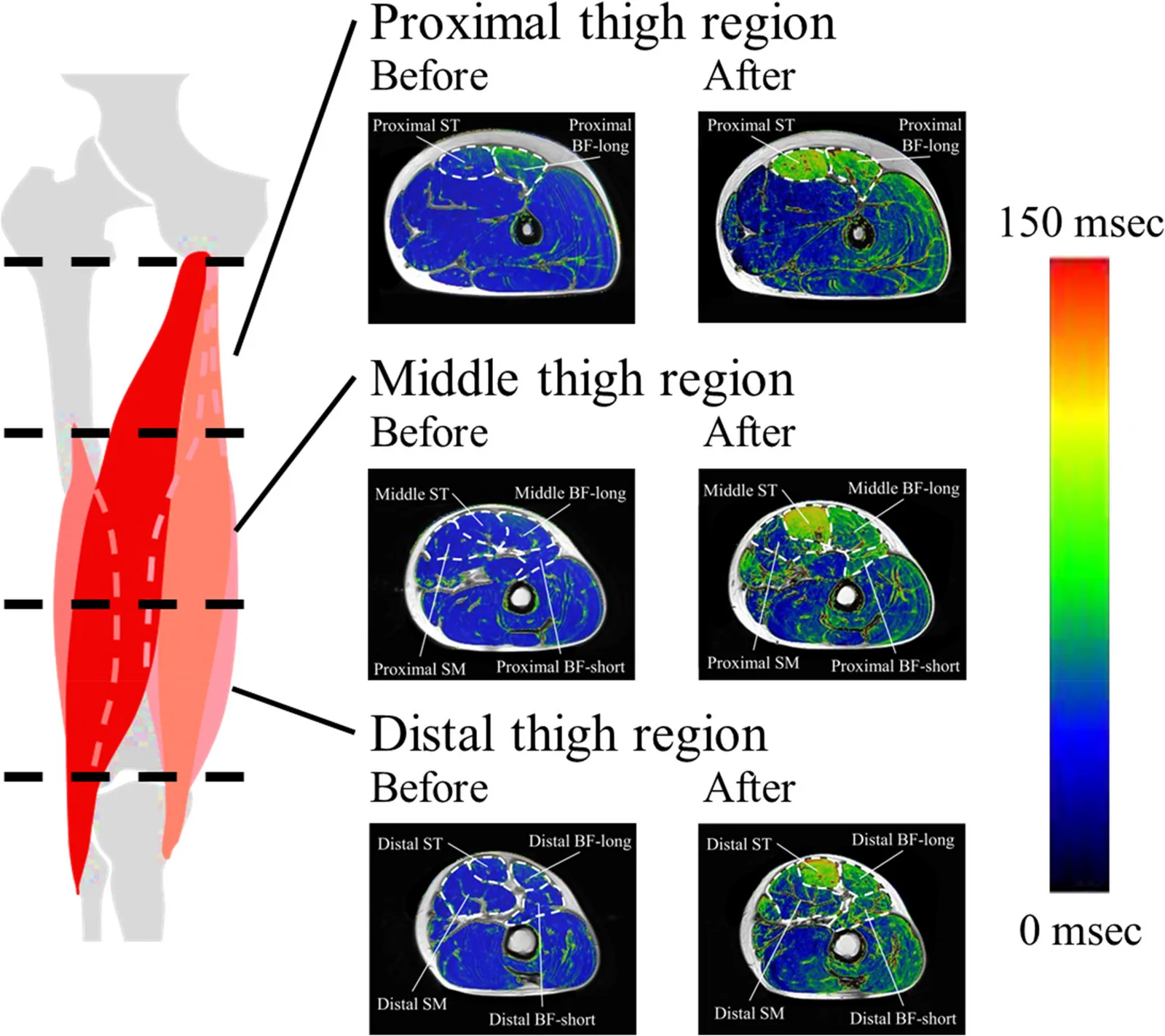
MRI slice segmentation and region-specific and longitudinally compartmentalized analysis of the hamstring. The 11 MRI slices were divided into three groups: four proximal, three middle, and four distal slices. Proximal thigh region: included the proximal parts of the BF-long and ST. Middle thigh region: included the middle parts of the BF-long and ST, as well as the proximal parts of the BF-short and SM. Distal thigh region: included the distal parts of the BF-long and BF-short, as well as the ST and SM. BF-long, biceps femoris long head; BF-short, biceps femoris short head; ST, semitendinosus; SM, semimembranosus

T2 values were calculated using dedicated imaging software (AZE Virtual Place AVP-001 A; Canon, Kanagawa, Japan). After superimposing the four echo-time images, the contours of the hamstring muscles were manually traced to define the region of interest in each image. Using the software, pixel-wise T2 values were computed, and mean T2 values for each slice were obtained. A pixel was considered activated by the exercise if its post-exercise T2 value exceeded the mean resting T2 value by more than one standard deviation, in accordance with a previously established criterion. The percentage of such activated pixels was calculated as the %T2 activation rate [34]. From the 25 acquired slices, 11 slices in which the hamstring muscles were clearly and consistently visible were selected for analysis. These 11 slices were selected for analysis because the muscle belly was consistently identifiable in all participants. The remaining slices were excluded, as a substantial proportion of the images consisted of tendon tissue. Each slice was analyzed twice, and the average of the two measurements was used as the final T2 value. The coefficient of variation between the two trials was 0.54 ± 0.35% (minimum: 0.07%, maximum: 0.97%), and the intraclass correlation coefficient ([1, 2]) was 0.996, indicating high reliability. The 11 selected slices were further divided into three longitudinal regions: four proximal slices (within a 7 cm range from the proximal myotendinous junction), three middle slices (within ± 2 cm of the midpoint of the femur: 50% of femoral length), and four distal slices (within a 7 cm range from the distal myotendinous junction), corresponding to the proximal, middle, and distal regions of the thigh, respectively. Average %T2 activation rates were calculated for each region [5]. The proximal thigh region included the proximal parts of the BF-long and ST; the middle thigh region included the middle parts of the BF-long and ST, along with the proximal parts of the BF-short and SM; and the distal thigh region included the distal parts of the BF-long and BF-short, as well as the ST and SM (Fig. 3). The number of muscles targeted in each region differs, however, this reflects an anatomical constraint arising from differences in muscle length among individual muscles. Therefore, it should be noted that the proximal thigh region does not include the BF-short or SM. Based on the computed %T2 activation rates, comparisons were made between the hamstring components. Furthermore, %T2 activation rates were compared across different HFA conditions within the same region and across different regions within the same HFA condition.

### Evaluation of subjective sensation of muscle contraction

The subjective sensation of hamstring muscle contraction was quantified using a numerical rating scale (NRS). The NRS was applied according to the method described by Mitsuya et al. and was defined on a scale from 0 to 10, where 0 represented “the weakest sensation of muscle contraction” and 10 represented “the strongest sensation of muscle contraction” [5]. Consistent with the T2 analysis, the thigh was divided into three regions: proximal, middle, and distal. Because it was difficult for participants to distinguish between the BF-long and BF-short, as well as between the ST and SM, the BF-long and BF-short were collectively classified as the lateral hamstring, whereas the ST and SM were classified as the medial hamstring for subjective evaluation. Accordingly, the hamstring was divided into six regions: (1) proximal lateral hamstring, (2) middle lateral hamstring, (3) distal lateral hamstring, (4) proximal medial hamstring, (5) middle medial hamstring, and (6) distal medial hamstring. NRS scores for each region were obtained verbally after each LC session consisting of 5 sets of 10 repetitions under the three HFA conditions.

### Statistical analysis

The Shapiro–Wilk test was used to assess normality. For comparisons of T2 values, Shapiro–Wilk test results indicated that the number of non-normally distributed datasets was small, and that sample sizes were equal across groups with only minimal variance imbalance. Therefore, the following statistical tests were conducted. Differences in 1RM among the three HFA conditions were analyzed using a one-way repeated-measures analysis of variance (ANOVA). To compare %T2 activation rates of the individual hamstring muscles, paired t-tests and two-way repeated-measures ANOVA were conducted, with muscle and HFA as within-subject factors for each thigh region. Additionally, differences in %T2 activation rates across different HFA within the same region, as well as across different regions within the same HFA, were analyzed using a two-way repeated-measures ANOVA with region and HFA as within-subject factors. When a significant main effect was observed, Bonferroni post hoc tests were applied. For the NRS analyses, the Wilcoxon signed-rank test was used for the Comparison of NRS Scores among the hamstring. The comparison of NRS scores between different HFA within the same hamstring region and the comparison of NRS scores among different hamstring regions within the same HFA condition were analyzed using the Friedman test. When a significant main effect was observed, post hoc comparisons were performed using the Wilcoxon signed-rank test with Bonferroni correction applied. Effect sizes for the ANOVA analyses were reported as partial η², whereas those for t-tests and post hoc comparisons were reported using Cohen’s d. The effect size for the Friedman test was reported as Kendall’s W, whereas the effect size for the Wilcoxon signed-rank test was reported as Cohen’s r. Cohen’s r was calculated as r=∣Z∣ / sqrt (N), where Z is the standardized test statistic and N is the number of observations included in the analysis. Effect sizes were interpreted as follows: for Cohen’s *d*, values < 0.2 were considered negligible, 0.2 ≤ *d* < 0.5 small, 0.5 ≤ *d* < 0.8 medium, and 0.8 ≦ d large. For Cohen’s r, values < 0.1 were considered negligible, 0.1 ≤ *r* < 0.3 small, 0.3 ≤ *r* < 0.5 medium, and 0.5 ≦ r large. All statistical analyses were performed using SPSS Statistics software (version 27.0.0.1 for Windows; IBM Corp., Armonk, NY, USA). Statistical significance was set at *p* < 0.05.

## Results

### LC 1RM

**Fig. 4:**
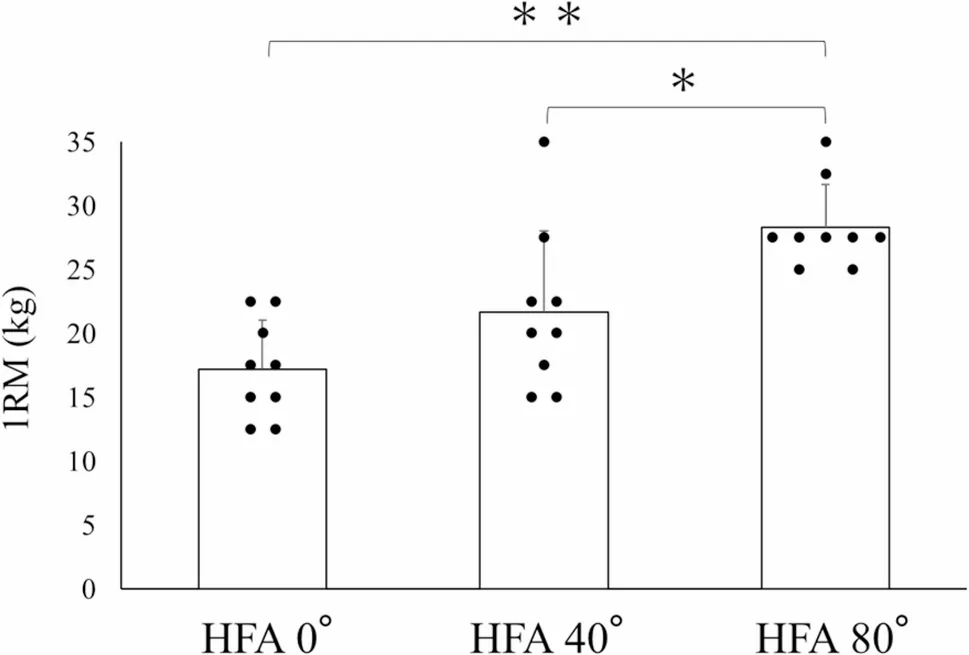
Comparison of LC 1RM among three HFA conditions. 1RM, one-repetition maximum; HFA, hip flexion angle. *: *p* < 0.05; **: *p* < 0.01

First, we compared LC 1RM among the three HFA conditions. A one-way repeated-measures ANOVA revealed a significant main effect (*p* < 0.001, partial η² = 0.515, Table 1). The 1RM in the HFA 80° condition was significantly higher than in the HFA 0° condition (*p* < 0.001, d = 3.119; Table 1, Fig. 4) and was also significantly higher than in the HFA 40° condition (*p* = 0.018, d = 1.296; Table 1, Fig. 4).

**Table 1:** Comparison of LC 1RM between three HFA conditions

HFA	0°	40°	80°
1RM (^kg^)	17.2 ± 3.8	21.7 ± 6.4*	28.3 ± 3.3**

Values are presented as mean ± standard deviation

*HFA* hip flexion angle, *1RM* one-repetition maximum

*: *p* < 0.05

**: *p* < 0.01

### MRI T2 activation rates

%T2 activation rates under each of the three HFA conditions are presented in Table 2 for each region.

**Table 2:** T2 activation rates of LC exercise between HFA and regions

(%)	BF-long			BF-short		ST			SM	
HFA	Pro	Mid	Dis	Pro	Dis	Pro	Mid	Dis	Pro	Dis
0°	61.5±20.3	53.5±20.6	48.8±18.9	54.2±28.1	54.2±25.1	69.8±25.2	74.3±21.2	76.1±19.3	47.1±20.8	44.9±24.6
40°	67.8±29.2	55.6±22.6	42.9±14.8	42.0±11.5	41.6±11.4	59.1±23.9	65.2±20.3	61.9±22.6	41.3±10.2	39.8±13.0
80°	52.1±19.8	49.1±15.1	45.4±14.0	41.2±12.2	46.0±20.3	55.1±10.4	59.1±13.1	54.5±19.8	44.4±15.8	43.2±17.0

Values are presented as mean ± standard deviation

*HFA* hip flexion angle, *BF-long* biceps femoris long head, *BF-short* biceps femoris short head, *ST* semitendinosus, *SM* semimembranosus, *Pro* proximal, *Mid* middle, *Dis* distal

### Comparison of VT2 activation rates within each hamstring

In the proximal thigh region, the %T2 activation rates of the proximal BF-long and proximal ST were compared. Paired t-tests revealed no significant differences under any of the HFA conditions (HFA 0°: *p* = 0.326, d = 0.363; HFA 40°: *p* = 0.347, d = 0.326; HFA 80°: *p* = 0.508, d = 0.190).

**Fig. 5:**
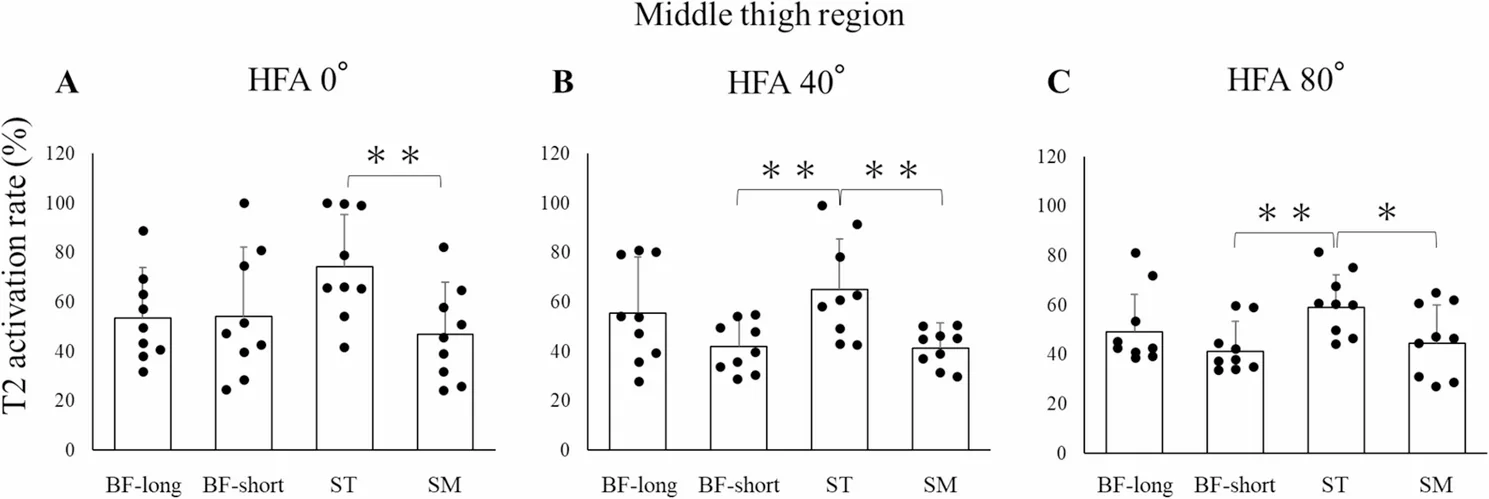
Comparison of %T2 activation rates among hamstring muscles in the middle thigh region. **A**, HFA 0° condition; **B**, HFA 40° condition; **C**, HFA 80° condition; HFA, hip flexion angle; BF-long, biceps femoris long head; BF-short, biceps femoris short head; ST, semitendinosus; SM, semimembranosus. *: *p* < 0.05; **: *p* < 0.01

In the middle thigh region, the %T2 activation rates of the middle BF-long, proximal BF-short, middle ST, and proximal SM were compared. A two-way repeated-measures ANOVA revealed significant main effects across all HFA conditions (HFA 0°: *p* = 0.009, partial η² = 0.154; HFA 40°: *p* < 0.001, partial η² = 0.234; HFA 80°: *p* = 0.002, partial η² = 0.195). At HFA 0°, the activation rate of the middle ST was significantly higher than that of the proximal SM (*p* = 0.010, d = 1.295; Fig. 5A). At HFA 40°, the middle ST showed significantly higher activation than both the proximal BF-short (*p* = 0.003, d = 1.406; Fig. 5B) and the proximal SM (*p* = 0.002, d = 1.448; Fig. 5B). At HFA 80°, the middle ST was significantly more activated than both the proximal BF-short (*p* = 0.002, d = 1.414; Fig. 5C) and the proximal SM (*p* = 0.016, d = 1.013; Fig. 5C).

**Fig. 6:**
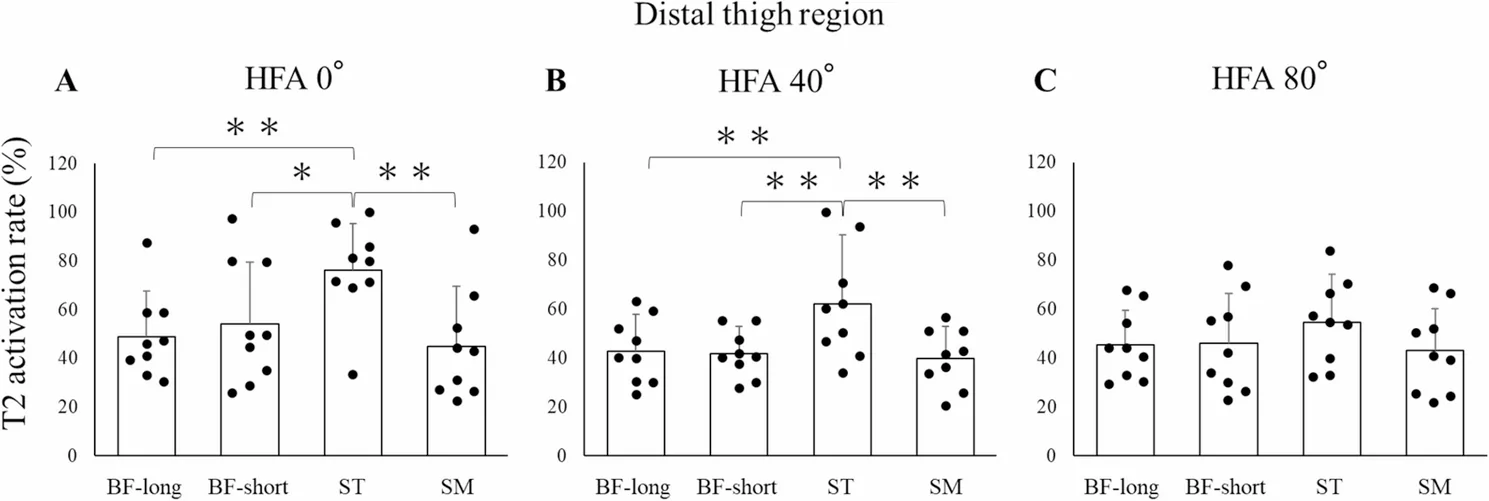
Comparison of %T2 activation rates among hamstring muscles in the distal thigh region. **A**, HFA 0° condition; **B**, HFA 40° condition; **C**, HFA 80° condition; HFA, hip flexion angle; BF-long, biceps femoris long head; BF-short, biceps femoris short head; ST, semitendinosus; SM, semimembranosus. *: *p* < 0.05; **: *p* < 0.01

In the distal thigh region, %T2 activation rates were compared among the distal BF-long, distal BF-short, distal ST, and distal SM. A one-way repeated-measures ANOVA revealed significant main effects at HFA 0° (*p* = 0.003, partial η² = 0.138) and HFA 40° (*p* < 0.001, partial η² = 0.203). At HFA 0°, the activation rate of the distal ST was significantly higher than those of the distal BF-long (*p* = 0.022, d = 1.429; Fig. 6A), distal BF-short (*p* = 0.012, d = 1.520; Fig. 6A), and distal SM (*p* = 0.006, d = 1.809; Fig. 6A). At HFA 40°, the distal ST was significantly more activated than the distal BF-long (*p* = 0.003, d = 0.995; Fig. 6B), distal BF-short (*p* = 0.001, d = 1.140; Fig. 6B), and distal SM (*p* < 0.001, d = 1.199; Fig. 6B). In contrast, no significant differences were observed among the muscles at HFA 80° (*p* = 0.146, partial η² = 0.057; Fig. 6C). These findings suggest that the ST tends to exhibit higher activation during LC exercises; however, under flexed hip positions, the activation levels of all hamstring muscles in the distal thigh appear to be uniform.

### Comparison of %T2 activation rates between different HFA within the same region

**Fig. 7:**
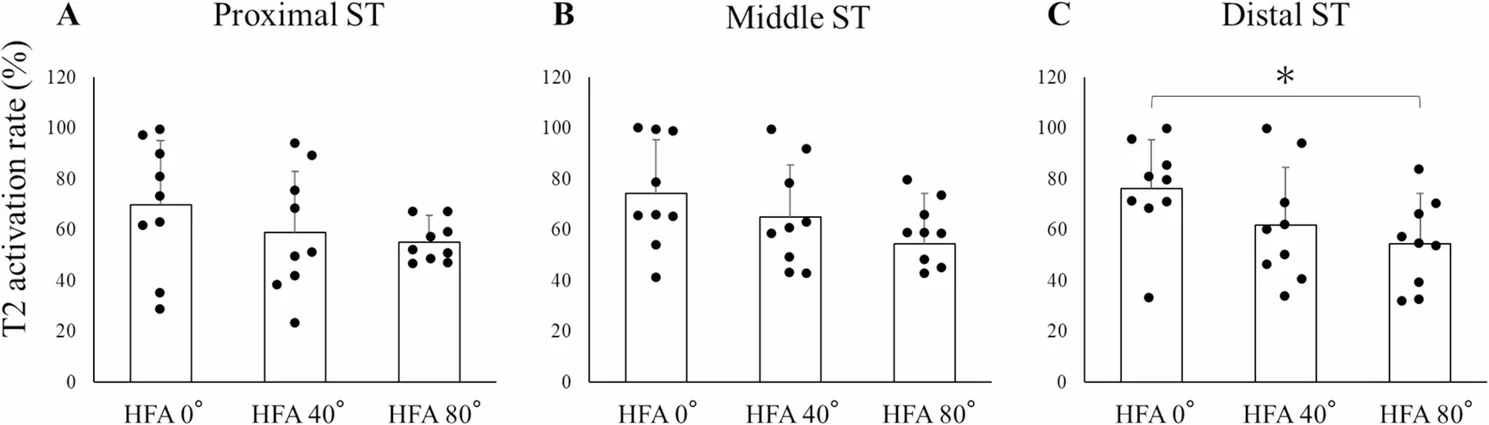
Comparison of %T2 activation rates across different HFA within the same region of the ST. **A**, proximal ST; **B**, middle ST; **C**, distal ST; HFA, hip flexion angle; ST, semitendinosus. *: *p* < 0.05

In the distal region of the ST, a significant main effect was observed (*p* = 0.024, partial η² = 0.103; Fig. 7C). Post hoc analysis revealed that the %T2 activation rate at HFA 0° was significantly higher than that at HFA 80° (*p* = 0.021, d = 1.105; Fig. 7C). In contrast, no significant differences were found in the proximal (*p* = 0.182, partial η² = 0.048; Fig. 7A) and middle (*p* = 0.105, partial η² = 0.085; Fig. 7B) regions of the ST. These findings suggest that decreasing the HFA—that is, moving toward a more extended position—may enhance activation in the distal ST during LC exercise.

### Comparison of %T2 activation rates between different regions within the same HFA

**Fig. 8:**
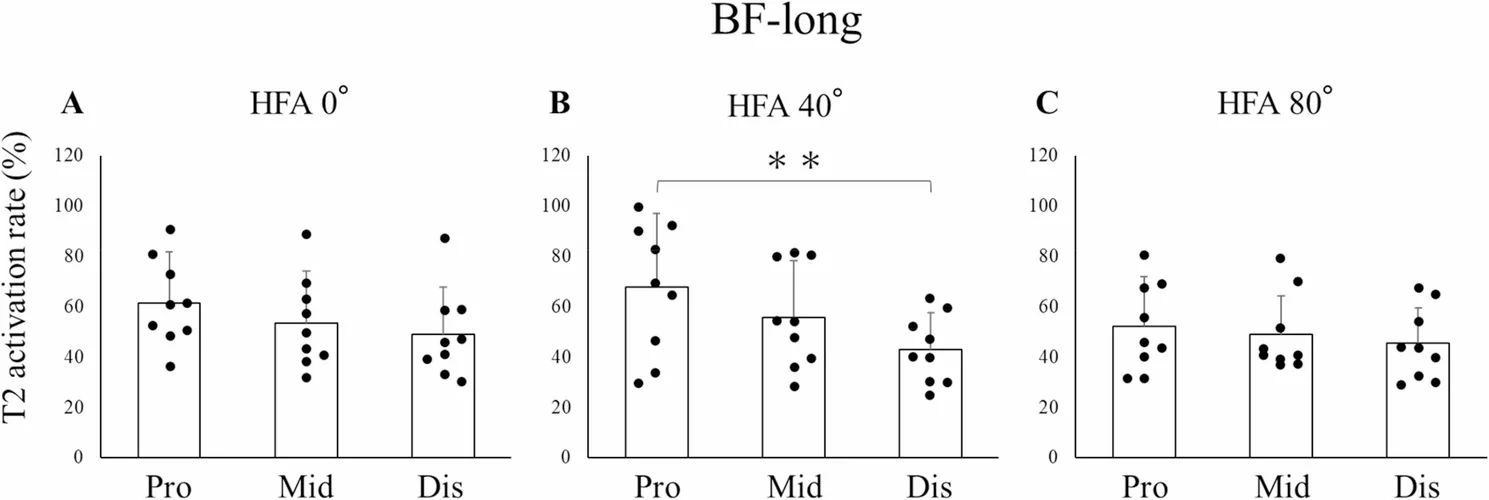
Comparison of %T2 activation rates among different regions of the BF-long within the same HFA. **A**, HFA 0° condition; **B**, HFA 40° condition; **C**, HFA 80° condition; HFA, hip flexion angle; BF-long, biceps femoris long head; Pro, proximal; Mid, middle; Dis, distal. **: *p* < 0.01

For the BF-long, a significant main effect was observed at HFA 40° (*p* = 0.002, partial η² = 0.182; Fig. 8B). Post hoc analysis revealed that the proximal BF-long exhibited significantly greater activation than the distal BF-long (*p* = 0.001, d = 1.076; Fig. 8B). No significant differences were found at HFA 0° (*p* = 0.462, partial η² = 0.117; Fig. 8A) or HFA 80° (*p* = 0.904, partial η² = 0.019; Fig. 8C). In contrast, no significant main effects or pairwise differences were observed for the BF-short (HFA 0°: *p* = 0.462, d = 0.000; HFA 40°: *p* = 0.904, d = 0.035; HFA 80°: *p* = 0.380, d = 0.287), ST (HFA 0°: *p* = 0.289, partial η² = 0.009; HFA 40°: *p* = 0.859, partial η² = 0.004; HFA 80°: *p* = 0.584, partial η² = 0.017), and SM (HFA 0°: *p* = 0.770, d = 0.097; HFA 40°: *p* = 0.685, d = 0.128; HFA 80°: *p* = 0.803, d = 0.073). These findings suggest that at HFA 40°, proximal BF-long activation is greater than that in the distal region, indicating longitudinally compartmentalized muscle activation within the same muscle.

### Comparison of NRS scores among the hamstring muscles

Table 3 presents the NRS scores for each hip flexion angle and region. To compare with T2 values, an objective index of muscle activity, NRS scores of the hamstring was analyzed as a subjective sensation of muscle contraction. Comparisons of NRS scores among the hamstring muscles revealed no significant differences in the proximal thigh region (HFA 0°: *p* = 1.000, *r* = 0.283; HFA 40°: *p* = 1.000, *r* = 0.272; HFA 80°: *p* = 1.000, *r* = 0.122), middle thigh region (HFA 0°: *p* = 0.054, *r* = 0.792; HFA 40°: *p* = 1.000, *r* = 0.000; HFA 80°: *p* = 0.123, *r* = 0.680), or distal thigh region (HFA 0°: *p* = 0.078, *r* = 0.744; HFA 40°: *p* = 1.000, *r* = 0.272; HFA 80°: *p* = 1.000, *r* = 0.280).

**Table 3:** NRS scores after LC exercise at each HFA and thigh region

	Numeric Rating Score					
	lateral hamstrings			medial hamstrings		
HFA	Proxmal	Medial	Distal	Proxmal	Medial	Distal
0°LC	5.4±2.2	5.7±1.7	3.4±2.6	5.8±3.2	7.8±1.2	4.7±2.9
40°LC	4.4±2.9	6.8±2.1	5.4±2.8	4.0±3.0	6.4±2.7	5.0±3.7
80°LC	3.2±2.5	4.7±1.9	5.9±2.5	3.3±2.5	5.8±1.2	6.3±2.4

The lateral hamstring consisted of the biceps femoris long head and biceps femoris short head, whereas the medial hamstring consisted of the semitendinosus and semimembranosus

Values are presented as mean ± standard deviation

*HFA* hip flexion angle, *LC* leg curl

### Comparison of NRS scores between different HFA within the same hamstring region

Friedman test revealed no significant main effects for the lateral hamstring (proximal: *p* = 0.345, Kendall’s W = 0.345; middle: *p* = 0.069, Kendall’s W = 0.420; distal: *p* = 0.495, Kendall’s W = 0.200) or the medial hamstring (proximal: *p* = 1.000, Kendall’s W = 0.111; middle: *p* = 0.084, Kendall’s W = 0.395; distal: *p* = 1.000, Kendall’s W = 0.040).

### Comparison of NRS scores among different hamstring regions within the same HFA condition

**Fig. 9:**
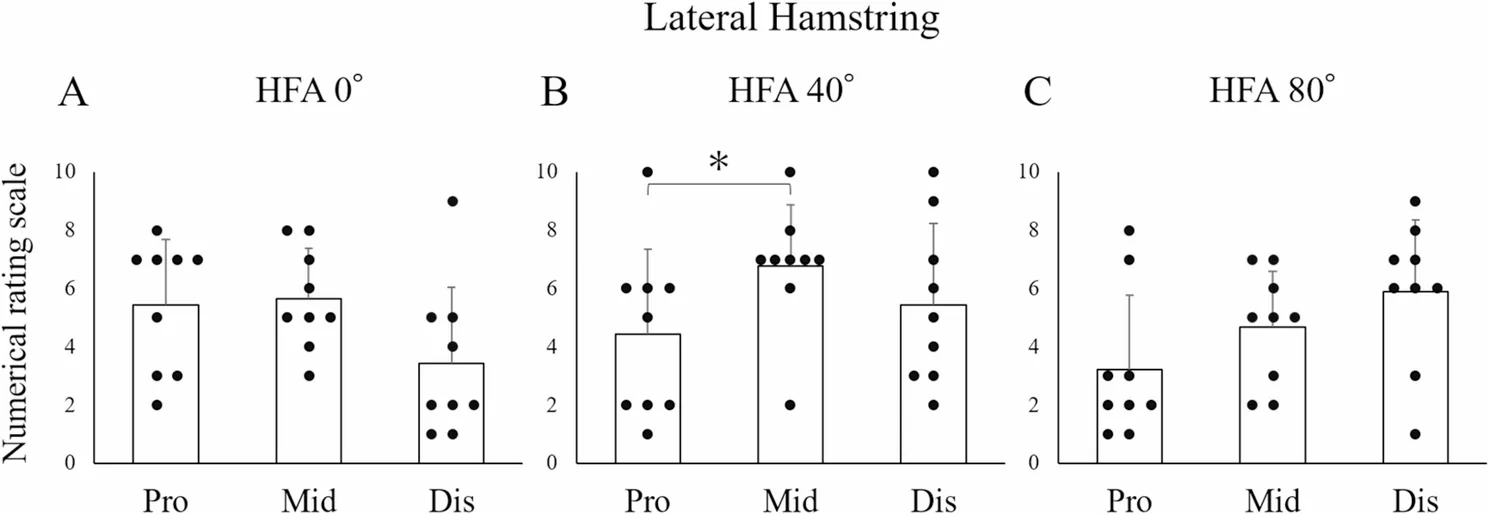
Comparison of NRS scores among different lateral hamstring regions within the same HFA condition. **A**, HFA 0° condition; **B**, HFA 40° condition; **C**, HFA 80° condition; HFA: hip flexion angle, *: *p* < 0.05

A significant main effect was observed in the lateral hamstring at HFA 40° (*p* = 0.045, Kendall’s W = 0.464). During LC at HFA 40°, the proximal lateral hamstring showed significantly lower NRS scores than the middle lateral hamstring (*p* = 0.048, *r* = 0.796; Fig. 9B). No significant differences were observed during LC at HFA 0° (*p* = 0.276, Kendall’s W = 0.265) or HFA 80° (*p* = 0.591, Kendall’s W = 0.181).

**Fig. 10:**
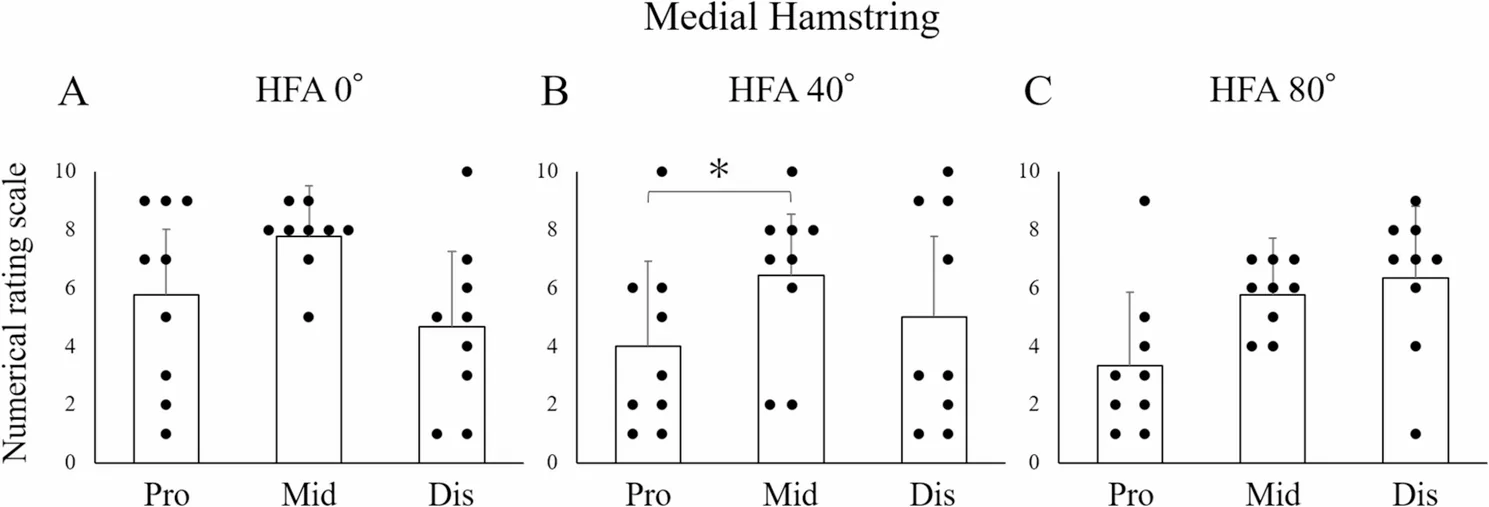
Comparison of NRS scores among different medial hamstring regions within the same HFA condition. **A**, HFA 0° condition; **B**, HFA 40° condition; **C**, HFA 80° condition; HFA: hip flexion angle, *: *p* < 0.05

A significant main effect was observed in the medial hamstring at HFA 40° (*p* = 0.048, Kendall’s W = 0.448). During LC at HFA 40°, the proximal medial hamstring showed significantly lower NRS scores than the middle medial hamstring (*p* = 0.011, *r* = 0.852; Fig. 10B). No significant differences were observed during LC at HFA 0° (*p* = 0.092, Kendall’s W = 0.265) or HFA 80° (*p* = 0.069, Kendall’s W = 0.758).

## Discussion

The purpose of this study was to determine whether the hamstring exhibit region-specific and longitudinally compartmentalized muscle dynamics by examining LC exercises performed at different HFAs. To this end, we assessed hamstring activation before and after LC exercises performed at three different HFAs: 0°, 40°, and 80°. These conditions represented, respectively, a fully extended hip position, a mid-range position, and a conventional seated LC position with near-vertical hip flexion. Using MRI to quantify T2 values, we analyzed activation across the proximal, middle, and distal regions of the hamstring. The results provided clear evidence of both region-specific and longitudinally compartmentalized activation patterns. At 1RM, HFA 80° showed higher values than HFA 0° and 40°. Across all conditions, the ST exhibited significantly greater activation compared with the other hamstring. In particular, at HFA 0°, the distal ST showed higher activation than the proximal ST. For the BF-long, greater activation was observed in the proximal than in the distal region at HFA 40°. In addition, the NRS scores were inconsistent with the objective measures. Based on these findings, the following discussion considers the implications of region-specific and longitudinally compartmentalized hamstring activation in relation to exercise execution and potential applications in resistance training practices. This study is novel in that it examined trained individuals performing LC exercises with precisely controlled HFA conditions, enabling detailed observation of region-specific muscle dynamics. Moreover, the use of well-trained bodybuilders with high proficiency and reproducibility in the exercise tasks, as well as the use of straps to minimize compensatory movements, represents a strength of this study.

Firstly, we discuss the region-specific activation patterns among the individual muscles comprising the hamstring. Previous studies have shown that different variations of the LC exercise elicit varying levels of activation and hypertrophy in the individual hamstring muscles [18, 35]. Therefore, to effectively and comprehensively strengthen the hamstring, it is essential to consider the activation characteristics of each constituent muscle. HFA-induced changes in hamstring length may alter the contribution of individual hamstring during the LC because of differences in their architectural characteristics. The BF-short and ST are classified as fusiform muscles, whereas the BF-long and SM are pennate muscles [14]. Because pennate muscles are generally capable of producing greater force than fusiform muscles [36], these architectural differences within the hamstring may have interacted with changes in muscle length to produce the observed region-specific responses. Previous research has reported that knee flexion force increases as HFA increases and hamstring length becomes longer [37]. Therefore, the relationship between muscle length and force production in muscles with different architectural characteristics may explain the region-specific muscle dynamics. In the present study, comparison of %T2 activation rates revealed a consistent trend of greater activation in the ST compared with other hamstring muscles. Buford et al. reported in cadaveric analyses that the ST contributed greater muscle tensile force during knee flexion than other hamstring muscles [38]. Indeed, the ST and BF-short have been reported to be preferentially activated during knee flexion exercises [39]. Furthermore, increased ST activation according to differences in exercise modality has been demonstrated using the same MRI-based methodology as that employed in the present study [40, 41]. Maeo et al. conducted 12 weeks of prone leg curl performed in a hip-extended position (approximately 20°) and seated leg curl performed in a hip-flexed position (approximately 80°) [18]. They reported that the ST exhibited significantly greater hypertrophy than the BF-short and SM, suggesting that the ST is preferentially activated during leg curl exercise. In contrast, the SM appears to have a narrower optimal length range than other hamstring muscles, with maximal torque and electromyographic activity observed near full knee extension and declining with progressive knee flexion [42–44]. A study comparing T2 values among 10 different hamstring exercises reported that the semimembranosus (SM) exhibited greater activation than the biceps femoris short head (BF-short) during the 45° hip extension exercise [40]. Consistent with these findings, the present study also showed relatively lower activation of the SM compared with other hamstring muscles. These results suggest that LC alone may not be sufficient to effectively activate or strengthen the SM. Therefore, incorporating exercises that emphasize hip extension, such as the deadlift, may be necessary to specifically target and strengthen the SM. However, electromyographic activity is not a direct indicator of muscle length and is strongly influenced by neural factors. Therefore, care should be taken not to confuse its interpretation with muscle tensile force.

Next, we discuss longitudinally compartmentalized muscle activation within the hamstring. Although previous studies have reported region-specific activation and hypertrophy patterns in the hamstring during LC exercises [18, 35], these investigations primarily focused on whole-muscle volume changes or utilized monopolar surface electromyography, without directly examining longitudinally compartmentalized activation. Studies focusing on the quadriceps femoris have demonstrated region-specific and longitudinally compartmentalized muscle dynamics during knee extension exercises performed at varying HFAs [5, 6]. These findings were attributed both to changes in muscle length resulting from alterations in HFA [5, 6], and to the branching of motor nerve innervations along the muscle’s longitudinal axis [13]. Similarly, in the hamstring, changes in muscle length due to variations in HFA, combined with the branching motor innervations in the BF-long, where motor nerve branches are divided between the proximal and distal regions [14], were hypothesized to underlie longitudinally compartmentalized activation. In our results, the %T2 activation rate in the distal ST was significantly higher during LC at HFA 0° compared with HFA 80° (Fig. 7), whereas activation across all hamstring muscles was comparable at HFA 80°. This suggests that when the hip is in a flexed position, ST activation is reduced, resulting in a more uniform activation pattern across the hamstring. In other words, longitudinally differentiated activation within the ST modulated the distribution of activity within the hamstring muscle group. These findings provide novel evidence that the hamstring—particularly the ST—exhibit longitudinally compartmentalized muscle activation, which may influence intramuscular recruitment patterns depending on joint position and exercise configuration. Using electromyography, Kubota et al. reported that the ST did not exhibit longitudinally compartmentalized muscle activation at an HFA 0°, the same HFA examined in the present study [15]. However, by applying the MRI-based methodology previously used to investigate longitudinally compartmentalized muscle dynamics in the quadriceps [5], we were able to observe these dynamics in the hamstring with greater precision. Previous studies have reported that effect sizes related to hamstring fascicle length vary considerably depending on ultrasound-based assessment methods, suggesting that different measurement techniques may capture different phenomena [45]. In the present study, MRI was used; therefore, it is considered that muscle dynamics were evaluated from a different perspective compared with ultrasound and electromyography [6]. While the BF-long and SM exhibit reduced activity during multi-joint exercises compared with single-joint exercises, the ST remains highly active under both conditions [4, 35]. One unique anatomical feature of the ST is the presence of a tendinous inscription near its midpoint [14], which may facilitate adaptive longitudinal compartmentalization across a range of movement tasks. Indeed, in the rectus abdominis, which contains multiple tendinous inscriptions, it has been reported that the upper and lower fibers exhibit different activation patterns depending on the exercise task [46]. Similarly, in the ST, which has a comparable anatomical structure, different fibers may be recruited depending on hip and knee joint angles, as well as whether the movement is hip or knee-dominant. Moreover, the ST is longer than the other hamstring components and generates force over a broader range of joint angles, making it less susceptible to reductions in torque or electromyographic activity associated with joint angle changes [15, 42]. These anatomical characteristics and patterns of muscle activity may contribute to a coordinated tension-control mechanism across the hamstring, facilitating precise regulation of muscle force. Future studies linking these anatomical and functional characteristics may further elucidate longitudinally compartmentalized muscle dynamics and their potential role in preventing muscle strain injuries.

The proximal BF-long showed greater activation at HFA 40°, and in our previous study focusing on the rectus femoris, activation was also higher at 40° compared with 0° and 80° [5]. These findings suggest that at HFA 40°, both the hamstring and the rectus femoris tend to exhibit stronger activation in their proximal regions. In previous studies, fascicle length of the BF-long has been reported to be the longest in a hip flexed and knee extended position, and the shortest in a hip extended and knee flexed position [47]. Furthermore, since higher knee flexion torque has been observed at an intermediate knee joint angle [21], it is suggested that both the hip and knee joints may approach an optimal muscle length in intermediate flexion–extension positions. At HFA 40°, both the hamstring and the rectus femoris may approach their optimal muscle length, allowing stronger activation even in the proximal region. In contrast, at HFAs 0° and 80°, the muscles are likely placed in excessively shortened or lengthened positions, which may limit their activation. Moreover, it has been reported that the proximal region of the BF-long is specifically activated during high-speed running [25]. Because the HFA 40° condition used in the present study corresponds to a hip intermediate position that frequently occurs during running [48], the preferential activation of the proximal BF-long observed at HFA 40° may be explained by this functional similarity. In addition, such region-specific loading on the proximal BF-long may contribute to the occurrence of proximal hamstring strain injuries [49]. These findings suggest that LC at HFA 40° may be a promising method for selectively loading the proximal hamstring, and its application for injury prevention should be further investigated in future longitudinal studies.

We discuss the results of the 1RM observed under the three HFA conditions. The 1RM at HFA 80° was significantly greater than those at HFAs 0° and 40°. This result is likely attributable to changes in hamstring muscle length induced by varying HFA, which in turn affected force production. Skeletal muscles generate less force when shortened or lengthened beyond their optimal length [50], suggesting that the hamstring may have been shortened relative to their optimal length in the more extended hip positions (0° and 40°), thereby reducing force output and resulting in lower 1RM values. In our previous study examining knee extension exercises at varying HFAs, no significant differences in 1RM were observed, indicating a contrasting trend to that seen in the present LC study [5]. This discrepancy may be partially explained by differences in muscle architecture. Fusiform muscles are more susceptible to changes in the length–tension relationship than pennate muscles. Specifically, the rectus femoris is a biarticular, pennate muscle, whereas the ST and BF-short are fusiform in structure [51, 52]. Therefore, architectural differences between muscles involved in LC versus knee extension may explain the divergent 1RM responses observed between the two exercise modalities. In particular, the force production of fusiform muscles such as the ST and BF-short may be more strongly influenced by changes in muscle length.

There have been no previous reports investigating subjective sensations of muscle contraction during LC exercise, despite their importance for selectively strengthening specific hamstring regions in practical resistance training settings. Therefore, the present study attempted to quantify subjective muscle contraction sensation using a NRS and to examine its relationship with objective muscle activity assessed by T2 values. Because participants found it difficult to distinguish between the long and short heads of the BF, as well as between the semitendinosus and semimembranosus, the BF-long and BF-short were collectively classified as the lateral hamstring, whereas the ST and SM were classified as the medial hamstring for subjective evaluation. As a result, during LC at HFA 40°, both the medial and lateral hamstring exhibited higher NRS scores in the middle region than in the proximal region. However, according to the T2 results, there was no condition in which the middle region showed the highest T2 activation rate in either the medial or lateral hamstring. These findings indicate that objective muscle activity and subjective sensations of muscle contraction in the hamstring do not necessarily correspond, even in resistance-trained individuals. In a previous study investigating leg extension performed at different HFAs, subjective sensations of muscle contraction and objective indices were consistent in the rectus femoris under the HFA 40° condition, whereas no such agreement was observed at the other joint angles (Mitsuya et al. 2023). In contrast, the present study found no such agreement in the hamstring under any LC condition. Subjective sensation of muscle contraction may reflect not only muscle activation itself, but also proprioceptive force perception and sensorimotor integration. For example, proprioceptive accuracy has been reported to decrease during force exertion in grip tasks [53]. In addition, proprioceptive accuracy has been shown to exhibit site-specificity [54]. Because proprioceptive responses differ between the anterior and posterior skeletal muscles of the lower extremity [55], subjective perception of muscle contraction may be less accurate in the hamstring than in the rectus femoris. Accordingly, when prescribing hamstring resistance training, it may be particularly important to understand objective muscle activation patterns rather than relying solely on subjective sensations of muscle contraction. Future studies are needed to further investigate why subjective sensations of muscle contraction did not correspond with objective indices in the hamstring, particularly from the perspective of neuromuscular activity relationships.

A primary limitation of the present study is its cross-sectional design, as it was not conducted as a longitudinal intervention. However, the utility of T2-weighted MRI for estimating muscle activation has been well established [34, 56–58]. Increases in T2 values are indicative of intramuscular fluid accumulation, which has been reported to occur following a single bout of resistance exercise and is thought to contribute to muscle hypertrophy [59]. Moreover, prior studies have shown that changes in %T2 activation rates correspond to both region-specific and longitudinally compartmentalized hypertrophy [7–9], supporting the relevance of %T2 activation rate increases as a proxy for muscle growth. Another limitation of this study is the relatively small sample size, and that the statistical power was not clearly defined. However, studies involving natural bodybuilders who regularly perform resistance training are extremely limited, and the present study provides valuable insights for understanding adaptations in muscle dynamics and trainability. Therefore, despite the small sample size, the significance of this study is considered to be substantial. Another limitation is that the study included only male bodybuilders and excluded female participants. The rationale for selecting this cohort was to obtain precise and reproducible data on the relationship between exercise execution and muscle dynamics, as experienced bodybuilders are highly proficient in controlling movement during resistance training. In addition, sex-related differences in the muscle fiber-type composition of the ST have been reported [60]. Future studies should aim to expand the participant pool to include female and untrained individuals to determine whether the present findings can be generalized to broader populations. In this protocol, a 72-hour interval was required between each measurement. Therefore, participants completed up to six testing sessions within a relatively short period (three 1RM tests and three LC sessions). Although a 72-hour recovery period was ensured, residual fatigue and training adaptations may have influenced subsequent sessions. However, since the order of trials was randomized, we consider that this potential effect was minimized as much as possible. Moreover, this limitation of the study is that detailed NRS data could not be obtained, and the hamstring was evaluated as a combined muscle group (lateral and medial hamstrings). Therefore, the comparison between the composite NRS and T2 values does not involve fully matched outcome measures. Accordingly, the comparison between NRS and T2 values should be interpreted with caution and only in a limited manner. Future studies should employ a more precise assessment of NRS to allow more rigorous comparisons.

## Conclusion

The ST exhibited greater activation than other hamstring muscles, particularly under more extended hip positions. Moreover, longitudinal differences in muscle activation were observed in both the BF-long and ST, depending on HFA. These findings provide novel evidence that hamstring activation is not only region-specific but also longitudinally compartmentalized, and that it is modulated by variations in HFA. Furthermore, because objective muscle activity did not correspond with subjective sensations of muscle contraction, caution should be exercised when relying on subjective perception in practical resistance training settings. Therefore, optimal hip positioning should be considered to effectively target specific hamstring regions in resistance training program design.

## Supplementary Information

Supplementary Material 1.

Supplementary Material 2.

## Data Availability

The datasets generated during the current study are available from the corresponding author upon reasonable request.

## Abbreviations

ANOVA: Analysis of variance
BF-long: Biceps femoris long head
BF-short: Biceps femoris short head
HFA: Hip flexion angle
MRI: Magnetic resonance imaging
NRS: Numeric rating scale
T2: Transverse relaxation time
SM: Semimembranosus
ST: Semitendinosus
LC: Leg curl
1RM: One—repetition maximum
